# Isolation and Antimicrobial Susceptibility of Bacteria from Chronic Suppurative Otitis Media Patients in Kerman, Iran

**Published:** 2011-12-01

**Authors:** K Mozafari Nia, G Sepehri, H Khatmi, M R Shakibaie

**Affiliations:** 1 Department of Otorhinolaryngology, Shafa Hospital, Kerman, Iran; 2 Department of Pharmacology, Neuroscience Research Center, Kerman, Iran; 3 Department of ENT, Physiology Research Center, Kerman, Iran; 4 Department of Microbiology, Kerman University of Medical Sciences, Kerman, Iran

**Keywords:** Chronic, Supportive, Otitis media, Antibacterial susceptibility, Iran

## Abstract

**Background:**

Chronic supportive otitis media (CSOM) is one of the commonest illnesses in ENT practice. This study was conducted to find out the various aerobic microorganisms associated with CSOM and their current antimicrobial susceptibility patterns to commonly used antimicrobials.

**Methods:**

samples were collected from 117 clinically diagnosed cases of CSOM and processed according to standard protocols.

**Results:**

Out of 117 CSOM cases, 105 (86%) showed positive bacterial culture. The Staphylococcus aureus was the commonest aerobic isolate in CSOM. The sensitivity of Staphylococci spp. to commonly used antimicrobials varied from 27.2% for cefixime to 95.5% for gentamicin and coagulase positive. Pseudomonas isolates showed complete (100%) resistance to amoxicillin/clavulanate (co-amoxiclave), cloxacillin and cefixime, and high sensitivity to ciprofloxacin (95%) and cephalexin (90%).

**Conclusion:**

An appropriate knowledge of antibacterial susceptibility of microorganisms would contribute to a rational antibiotic use and the success of treatment for chronic supportive otitis media.

## Introduction

Chronic supportive otitis media (CSOM) is the chronic inflammation of the middle ear and mastoid mucosa in which the tympanic membrane is perforated and discharges of grayish-white, homogeneous, turbid, and viscous secretions are present.[1][2][3] CSOM most often occurs in the first 5 years of life, and is common in developing countries, in special populations such as children with craniofacial anomalies and in certain racial groups.[2][4]

The aerobic microorganisms most frequently isolated in CSOM are Pseudomonas aeruginosa, Staphylococcus aureus, Gram-negative organisms such as Proteus spp., Klebsiella spp., and Escherichia spp., Haemophilus influenzae, and Moraxella catarrhalis.[1][3][5] The most frequently isolated anaerobic organisms were Bacteroides spp. and Fusobacterium spp.[5][6]

Since the bacteriology and antimicrobial susceptibility of CSOM infections were not determined in Iran, this study was performed to evaluate the antimicrobial susceptibility patterns among aerobic bacteria isolated from CSOM patients in ENT clinics in Kerman, Iran.

## Materials and Methods

Samples were collected from 117 clinically diagnosed cases of CSOM by ENT specialists at private clinics and ENT educational clinics of Kerman University of Medical Sciences in Iran and processed according to standard protocols. CSOM was defined as otorrhea through a perforated tympanic membrane present for at least 2-6 weeks.[2][3] Exclusion criteria were current febrile illness, current antibiotic use or use in the preceding 2 weeks, need for renal dialysis, recent ear surgery or an in-situ grommet or tympanostomy tube, mastoid surgery in the preceding 12 months, congenital ear or hearing problems, obstructed middle ear (eg, polyp) and pregnancy.

An ear swab was obtained by inserting a sterile swab deep in the ear canal and the discharges were added to Stuart transport medium and transported to a microbiology test laboratory. Organisms were identified using standard methods and API identification system (bioMérieux, Basingstoke, UK).[7]

Gram positive and gram-negative bacterial sensitivity of isolates to commonly used antimicrobials (gentamicin, ciprofloxacin, amoxicillin/clavulanate, (co-amoxiclave), cloxacillin, cefixime and cephalexin) were investigated by disk diffusion method using NCCLS guidelines.[8] Data were analyzed by SPSS software (Version 16, Chicago, IL, USA).

## Results

The culture samples of the 105 out of 117 patients were positive, yielding 128 bacteria. Fungi were isolated in 21 patients (24.57%) and 12 patients (14.04%) had neither bacteria nor fungi infections (Table 1). Staphylococci species (50.3%) were the most prevalent microorganisms isolated followed by Pseudomonas aeruginosa (23.4%) (Table 1).

**Table 1:** The bacteriological findings obtained from 117 CSOM^a^ patients

**Species**	**No. of isolates**	**% of isolates**
No growth	12	14.04
Coagulase (+) Staphylococcus	22	25.74
Coagulase (-) Staphylococcus	21	24.57
Pseudomonas aeruginosa	20	23.40
Escherichia coli	7	8.19
Streptococcus	6	7.02
Proteus	5	5.85
Klebsiella	5	5.85
Enterococcus	3	3.51
Citrobacter	2	2.34
Enterobacter	1	1.17
Fungi	21	24.57
Mixed Infection	11	12.9

^a^ CSOM: Chronic suppurative otitis media.

Table 2 shows the resistance rates of the main isolated pathogens (Staphylococci species and Pseudomonas aeruginosa, proteus and Klebsiella) from CSOM patients to commonly prescribed antimicrobials. The co-infection with several bacterial species (including Staphylococcus aureus and Pseudomonas aeruginosa) was seen in 11 (12.9%) patients (Table 1).

**Table 2:** Antibiotic susceptibility of isolated microorganisms from otorrhea in 117 CSOM patients to commonly used antimicrobials^a^.

**Species**	**CP**	**CFM**	**CO-AMOX**	**CLOX**	**GM**	**CF**
Coagulase (+) Staph	85.4	27.2	63.6	81.8	95.5	90.9
Coagulase (-) Staph	52.4	33.3	52.4	57.1	47.6	57.1
Pseudomonas	95	0	0	0	85	5
Proteus	80	60	0	0	60	40
Klebsiella	60	20	80	20	60	40

^a^ CSOM: Chronic suppurative otitis media. CP: Ciprofloxacin, CFM: Cefixime, CO-AMOX: Amoxicillin/Clavulanate (Co-amoxiclave), CLOX: Cloxacillin, GM: Gentamicin, CF: Cephalexin.

Sensitivity of coagulase negative Staphylococci spp. to commonly used antimicrobials varied from 33.3% for cefixime to 57.1% for cloxacillin and cephalexin (Table 2). Pseudomonas isolates showed complete (100%) resistance to amoxicillin/ clavulanate (co-amoxiclave), cloxacillin and cefixime, but it showed high sensitivity to ciprofloxacin (95%) and cephalexin (95%) (Table 2). Proteus spp. showed relatively high sensitivity to ciprofloxacin (80%) and gentamicin (60%). Also co-amoxiclave, ciprofloxacin and gentamicin showed good antibacterial activity against Klebsiella spp. (Table 2).

## Discussion

The results of this study showed that Staphylococcus aureus was the commonest aerobic isolate in CSOM followed by Pseudomonas aeruginosa which is in agreement with the reports of some other investigators in different parts of the worlds, however, others reported that Pseudomonas aeruginosa was the commenest isolated microorganism in CSOM patients.[1][3][5][9]

Streptococcus, Proteus, Klebsiella, Enterococcus, Citrobacter, Enterobacter and fungi were isolated in some CSOM patients which is comparable to the results of other investigators.[5][6] The sensitivity of coagulase negative Staphylococci spp. to commonly used antimicrobials varied from 27.2% for cefixime to 57.1% for cloxacillin and cephalexin .However, coagulase positive Staphylococci spp. were more sensitive to commonly used antimicrobials and showed high sensitivity rates to gentamicin (95.5%), cephalexin (90.9%) and ciprofloxacin (85.4%). Clinical resistance of Staphylococci spp. to penicillin and other antimicrobial agents is now a problem throughout the world.[10][11][12]

Staphylococci spp. sensitivity to ciprofloxacin is in agreement with other reports and most of the investigators reported high sensitivity rate for Staphylococci spp. to fluoroquinolones such as ofloxacin and ciprofloxacin.[5][10][13] Pseudomonas isolates showed complete (100%) resistance to amoxicillin/clavulanate (coamoxiclave), cloxacillin and cefixime which is in contradictory to other reports, although some other investigators showed high resistance rate for Pseudomonas isolates to beta-lactam antibiotics.[1][5][6][14]

Pseudomonas showed high sensitivity to ciprofloxacin (95%) and it was relatively sensitive to gentamicin (85%). High fluoroquinolones antibacterial activity against Pseudomonas isolates was reported by others, although resistant strains of Pseudomonas isolates to fluoroquinolones were detected in other studies.[1][5][10][13]

Coagulase positive Staphylococci and Pseudomonas showed high sensitivity to gentamicin which is comparable to the results of Gul et al. (58%).[1][15] Others reported a relatively low bacterial resistance to both coagulase positive Staphylococci and Pseudomonas isolates.[14] The resistance to commonly used antimicrobials in Iran has been reported by other investigators too.[16][17][18]

In summary, the results of this study showed high resistance rate of Staphylococci and Pseudomonas isolates from CSOM patients to ß- lactam and other commonly used antimicrobials. Therefore, an appropriate knowledge of antibacterial susceptibility of microorganisms may contribute to rational antibiotic use and the success of treatment for chronic supportive otitis media.
